# Hemolytic Uremic Syndrome: A Case Report

**DOI:** 10.31729/jnma.8151

**Published:** 2023-05-31

**Authors:** Sandesh Gaire, Mandira Shrestha, Chaitanya Darshan Bhattarai, Susajjan Dhungana, Surakshya Gyawali, Arun Bajgain

**Affiliations:** 1 Nepal Medical College and Teaching Hospital, Jorpati, Kathmandu, Nepal; 2 Department of Paediatrics, Nepal Medical College and Teaching Hospital, Jorpati, Kathmandu, Nepal

**Keywords:** *anemia*, *case reports*, *dehydration*, *renal replacement therapy*

## Abstract

Thrombotic microangiopathy is a pathological condition comprised of microvascular thrombosis involving any body organ leading to thrombocytopenia, coombs-negative hemolytic anemia, and end-organ damage. The clinical presentation of the case shows typical hemolytic uremic syndrome, however, lab reports show atypical hemolytic uremic syndrome (low C3). Pain abdomen and loose stool with some signs of dehydration were initial presentations. Early initiation of renal replacement therapy and management of dehydration was done. Simple diarrhea can also manifest as acute kidney injury with the hemolytic uremic syndrome. Hence we should keep hemolytic uremic syndrome as the differential diagnosis of diarrhea. Irrespective of lab parameters, early management in line with the typical hemolytic uremic syndrome should be done for better outcomes.

## INTRODUCTION

Thrombotic microangiopathy is a condition comprising microvascular thrombosis leading to thrombocytopenia, coombs-negative hemolytic anemia, and end-organ damage. The most common forms are shiga toxin-producing *Escherichia coli* mediated hemolytic uremic syndrome (HUS), thrombotic thrombocytopenic purpura, and atypical HUS.^1^ The incidence of HUS among children <15 years is 1.44 per 100,000.^2^ A triad of renal insufficiency, thrombocytopenia, and hemolytic anemia affects about 50% of cases.^3^ *E. coli* 0157:H7-related acute gastroenteritis is regularly linked to HUS, which typically manifests after prodromal bloody diarrhea.^4^ We present a case of a 12-year-old female with a history of microangiopathic hemolytic crisis, abdominal pain, and diarrhea.

## CASE REPORT

A 12-year-old female presented to our hospital emergency with the chief complaint of abdominal pain for 6 days and loose stool for 6 days. Abdominal pain was acute in onset, initially in, a periumbilical region which then progressed to all areas of the abdomen, pricking in nature, moderate to severe in intensity which was accompanied by loose stool. The loose stool was 6-7 times a day, watery in consistency. On the second day of illness, the loose stool was accompanied by blood, bright red in color. The patient had nausea and occasional vomiting after every feed. The patient also had a history of reddish skin rashes starting from hands and gradually progressing towards the whole body 3 days before the day of illness which was relieved by taking medication. The patient had a history of taking food something out of ordinary. With the above complaints, the patient was taken to a nearby clinic where she was prescribed some medications; then the patient was taken to a tertiary center for treatment and was admitted as a case of the acute abdomen on investigation.

Complete blood count showed decreased platelet count (93000) and stool R/E was positive for occult blood with plenty red blood cells (RBC). Renal function test showed increased Urea (33.5 mg/dl) and creatinine (1.1 mg/dl), so with these investigations and symptoms in mind, they sent the child for further management to a higher center as a suspected case of HUS with acute kidney injury (AKI) after 1 day of admission and was advised for pediatric intensive care unit (PICU) admission and need for renal replacement therapy (RRT) for which she was referred to our center. On the day of presentation at our center, a physical examination revealed a lethargic, ill-looking child with some signs of dehydration and normal body temperature, blood pressure of 110/80 mm of Hg, heart rate of 106 beats/min, respiratory rate of 24 breaths/min with no signs of distress and oxygen saturation by pulse oximetry of 93% in room air with a capillary refill time of fewer than 3 seconds. Her weight was 36 kg with a height of 151 cm. On head-to toe examination, it revealed periorbital edema and bilateral pitting edema up to the knee. Systemic examination revealed normal cardiac and chest findings, except for tachycardia, the abdomen was distended, and diffuse tenderness was present with no organomegaly.

Laboratory tests showed: hemoglobin (Hb) 8.5 g/dL, platelet count 55x10^3^/uL, white blood cell (WBC) count 7700/cumm, blood urea 195 mg/dL, blood creatinine 5.9 mg/dl lactate dehydrogenase (LDH) 3289 U/L, peripheral blood smear showed predominantly normocytic normochromic cell admixed with few polychromatophils and schistocytes. Urinalysis revealed haematuria (plenty of RBC) and proteinuria (albumin +++). Her stool routine examination showed RBC 0-3/hpf, pus cells 1-3/hpf, and was positive for occult blood. The stool culture showed no growth(she was already 3 days of antibiotics). Her basic metabolic panel revealed a sodium level of 138 mg/dL, a potassium level of 4.2 mg/dL, and a bicarbonate level of 15.08 mmol/L. For further evaluation, an abdomen ultrasound was done, which showed a slightly echogenic bilateral kidney with maintained corticomedullary differentiation, with moderate ascites.

With above mentioned clinical symptoms and laboratory findings, the patient was suspected as a case of typical HUS with AKI and admitted to the ICU. The patient was then taken for hemodialysis via the central line (internal jugular vein) and further diagnostic investigations were carried out to rule out other causes of hemolysis and acute kidney injury, which included serum anti-nuclear antibody, direct coombs test, serology, complement C3 (low), which all other tests within the normal limit.

During the course of admission, the patient was subjected to multiple transfusions as Hb was 4.5 mg/dl (4 pints of blood with one-pint equalling 350 ml) and underwent hemodialysis (6 times). The patient's urea and creatinine came to the normal limit after sixth hemodialysis.

During her hospital stay, she was first treated with intravenous fluid therapy in the view of pre-renal AKI however, the value of creatinine was on the rising trend so the patient was subjected to fluid restriction. She was transferred out from ICU after 4 days. She received antibiotic therapy which included Inj ceftriaxone 2g/24hr (renal adjusted dose) for 8 days and Inj Metronidazole 800mg/24hr (renal adjusted dose) for 8 days. After day 8 she was kept on tablet cefixime 130g for 5 more days. Laboratory studies at discharge included a hemoglobin of 10.5 g/dL, platelets of 313000, blood urea of 35 mg/dL, and serum creatinine of 1 mg/dL. After 17 days of hospital stay, she was discharged and received outpatient follow-up care.

## DISCUSSION

HUS is a rare condition characterized by progressive renal failure, thrombocytopenia, and hemolytic anemia. It can be classified as either typical (diarrhea-associated) or atypical (non-diarrhea associated such as following a urinary tract infection)^5^. Originally, Hemolytic Uremic Syndrome (HUS) was classified based on the presence of diarrhea, as Diarrhoea Positive HUS (D+HUS) and diarrhea negative HUS (D-HUS). However this has changed in recent times, Now HUS is classified, clinically as Primary HUS, and secondary HUS. Primary HUS is also referred to as atypical HUS, or the older Diarrhoea negative HUS, in which HUS due to complement deregulation. The more common secondary HUS, or typical HUS, is further divided into infectious and non-infectious causes. The most common cause of HUS is Shiga toxin-producing Escherichia coli (STEC) (O157; H7)most commonly found in undercooked beef and it is one of the main causes of acute kidney injury in children under the age of 5 years6. Diagnosing Hemolytic Uremic Syndrome (HUS) is done clinically, based on the classical triad of microangiopathic hemolytic anemia, thrombocytopenia, and acute kidney injury. HUS can be established by laboratory examination, such as a complete blood count, with differential, and peripheral blood smear, renal functions studies, and urinalysis. The microangiopathic hemolytic anemia in HUS can be confirmed with hemoglobin and a hematocrit level, and this anemia is typically coombs negative in character^6^. Our patient also presented with diarrhea and lab investigation showed thrombocytopenia(schistocytes>12% which is significant), and decreased hemoglobin with a deranged renal function test . The Coombs test was negative too but the complement level was low(which is a feature of atypical HUS). Typical hemolytic uremic syndrome (HUS) is a leading cause of community-acquired acute kidney injury in infants and young children.^7^ our patients went through hemodialysis 4 times due to AKI.

Currently, there is no evidence that treatments other than supportive care, including dialysis and ventilator support as required, improve the outcome of patients with STEC HUS. Plasma-based therapy is no longer recommended for the treatment of STEC HUS and may in fact be harmful.
